# Activity-based protein profiling guided identification of urine proteinase 3 activity in subclinical rejection after renal transplantation

**DOI:** 10.1186/s12014-020-09284-9

**Published:** 2020-06-16

**Authors:** Mario Navarrete, Brice Korkmaz, Carla Guarino, Adam Lesner, Ying Lao, Julie Ho, Peter Nickerson, John A. Wilkins

**Affiliations:** 1 Manitoba Centre for Proteomics and Systems Biology, 799 John Buhler Research Centre, 715 McDermot Ave., Winnipeg, MB R3E3P4 Canada; 2 INSERM, UMR 1100, “Centre d’Etude des Pathologies Respiratoires”, Université de Tours, 37032 Tours, France; 3 Faculty of Chemistry, University of Gdansk, 80-308 Gdansk, Poland; 4 Section Biomedical Proteomics, Dept. Internal Medicine, University of Manitoba, Winnipeg, MB Canada; 5 Section of Nephrology, Dept. Internal Medicine, University of Manitoba, Winnipeg, MB Canada; 6 Dept. Immunology, University of Manitoba, Winnipeg, MB Canada

**Keywords:** ABPP/activity-based protein profiling, Urine, PR3/PRTN3/myeloblastin, Renal transplant, Serine hydrolase, ABHD14B/CCG1-interacting factor B, LTF/LactotransferrinPR3, Neurotrypsin/PRSS12

## Abstract

**Background:**

The pathophysiology of subclinical versus clinical rejection remains incompletely understood given their equivalent histological severity but discordant graft function. The goal was to evaluate serine hydrolase enzyme activities to explore if there were any underlying differences in activities during subclinical versus clinical rejection.

**Methods:**

Serine hydrolase activity-based protein profiling (ABPP) was performed on the urines of a case control cohort of patients with biopsy confirmed subclinical or clinical transplant rejection. In-gel analysis and affinity purification with mass spectrometry were used to demonstrate and identify active serine hydrolase activity. An assay for proteinase 3 (PR3/PRTN3) was adapted for the quantitation of activity in urine.

**Results:**

In-gel ABPP profiles suggested increased intensity and diversity of serine hydrolase activities in urine from patients undergoing subclinical versus clinical rejection. Serine hydrolases (n = 30) were identified by mass spectrometry in subclinical and clinical rejection patients with 4 non-overlapping candidates between the two groups (i.e. ABHD14B, LTF, PR3/PRTN3 and PRSS12). Western blot and the use of a specific inhibitor confirmed the presence of active PR3/PRTN3 in samples from patients undergoing subclinical rejection. Analysis of samples from normal donors or from several serial post-transplant urines indicated that although PR3/PRTN3 activity may be highly associated with low-grade subclinical inflammation, the enzyme activity was not restricted to this patient group.

**Conclusions:**

There appear to be limited qualitative and quantitative differences in serine hydrolase activity in patients with subclinical versus clinical renal transplant rejection. The majority of enzymes identified were present in samples from both groups implying that in-gel quantitative differences may largely relate to the activity status of shared enzymes. However qualitative compositional differences were also observed indicating differential activities. The PR3/PRTN3 analyses indicate that the activity status of urine in transplant patients is dynamic possibly reflecting changes in the underlying processes in the transplant. These data suggest that differential serine hydrolase pathways may be active in subclinical versus clinical rejection which requires further exploration in larger patient cohorts. Although this study focused on PR3/PRTN3, this does not preclude the possibility that other enzymes may play critical roles in the rejection process.

## Background

Standard-of-care kidney transplant monitoring strategies are limited in their ability to detect ongoing rejection as clinical markers only detect loss of graft function [1, 2]. Indeed, subclinical rejection is a smouldering rejection phenotype that is only detectable by surveillance biopsies and is associated with preserved graft function [3]. Subclinical T-cell mediated rejection (TCMR) is an important predictor of late graft failure [4–9]; and its treatment results in improved histology [10, 11], with similar graft survival compared to patients without subclinical TCMR [12]. Taken together, these observations demonstrate that subclinical rejection is a clinically significant and treatable form of autoinflammation.

Current transplant paradigms suggest that subclinical rejection is the same process as clinical rejection but simply at an earlier stage [13]. However, untreated subclinical rejection does not universally evolve to clinical rejection [14] suggesting potential underlying differences. Furthermore, subclinical rejection has a unique transcriptome compared to early clinical TCMR, suggesting that differing molecular processes may lead to infiltrating T-cells on biopsy [15]. Overall, the pathophysiology of subclinical versus clinical rejection remains incompletely understood given their equivalent histological severity yet highly discordant presentation in graft function.

Transcriptomic, proteomic and metabolomic approaches have been used to characterize kidney transplant rejection and identify potential biomarkers for subclinical rejection [16–20]. However, the detection of a protein does not necessarily imply functional activity. Enzymes are often synthesised in a latent state requiring post-translational processing to generate the active form of the molecule. Detection methods based on mass spectrometry or immunoassays generally do not provide information on the functional status of enzymes. The development of activity-based protein profiling (ABPP) in conjunction with mass spectrometry offers a relatively unbiased mechanism-based approach to identifying active members of enzyme families in complex biological samples [21, 22]. Analysis of protein activities may be more physiologically relevant than quantitative analysis, and also offers a basis for developing real-time quantitative assays. Evaluating protein functional states may help characterize the underlying biochemical processes during rejection [23]. The goal of the present study was to use ABPP to identify and compare the active serine hydrolase enzymes in urine during subclinical and clinical rejection to further our understanding of their underlying pathophysiology in kidney transplant patients.

## Methods

### Study population

A prospective single center cohort of adult kidney transplant patients had urines collected in conjunction with surveillance or clinical indication biopsy for proteomic analysis. This cohort has been previously described, the protocol was approved by the University of Manitoba REB (ethics number HS15993, H2013:017) and informed consent obtained from all participants [18]. The groups were defined as:

1. Healthy individuals (n = 20): normal urines from healthy volunteers for assay development.
2. Normal transplant (n = 6): Stable graft function and normal surveillance biopsies, Banff acute scores i0 t0 v0 g0.
3. Subclinical rejection (n = 6): Banff acute scores: i ≥ 2 t ≥ 2 v0 g0, Banff chronic scores: ci0 ct0 ± cv > 1 cg0, stable graft function, and surveillance biopsy.
4. Clinical rejection (n = 6): Banff acute scores: i ≥ 2 t ≥ 2 v0 g0, Banff chronic scores: ci + ct ≤ 1 ± cv > 1 cg0, and clinical indication biopsy for decline in graft function.

### Activity probe labelling of urine serine hydrolases

Urine samples were adjusted to pH 9.0 with concentrated Tris to a final concentration of 50 mM and reacted with 2 μM Fluorophosphonate (FP)-TAMRA (Product number 88318, Thermo Scientific, Rockford IL USA) or 0.5 mM 6-*N*-biotinylaminohexyl isopropyl phosphorofluoridate, PF-biotin (Product number B394900, Toronto Research Chemicals, North York ON Canada). The samples were incubated with the probe for 90 min at 37 °C and the reaction was stopped by adding 4× SDS sample buffer (Product number NP0007, Life Technologies, Carlsbad CA USA) and heating for 5 min at 95 °C in the presence of 50 mM DTT. Proteins were then separated on Bolt 4–12% Bis–Tris plus SDS-PAGE gels (Product number NW04120BOX, Life Technologies, Carlsbad CA USA) at 120 volts, 80 min. Gels were washed in distilled water for 20 min before scanning fluorescence at 534 nm to visualize labelled proteins. Then same gels were overnight incubated with 100 mL SYPRO™ Ruby protein gel stain (product number S12001, Thermo Fischer scientific) then washed with 10% methanol, 7% acetic acid solution for 30 min before scanning for fluorescent at 534 nm or gels were incubated with Coomassie blue (Gel code, product number 24590, Thermo Fischer scientific), washed with water and photographed.

### Affinity purification of activity probe-labeled serine hydrolases

Initial experiments were performed using two 6 mL pools each (1 mL each from 6 patients per group) from either subclinical or clinical rejection patients. One pool from each group was labelled with either FP TAMRA or PF Biotin. Subsequently purification of serine hydrolases was done from two 12 mL pooled urine samples generated from either subclinical or clinical rejection patients using 2 mL from 6 patients per group. Pools were 0.22 μm syringe-filtered (Product number SLGV033RS, Millipore, Tullagreen, Carrigtwohill, CORK IRL) and concentrated 25 fold with a centrifugal filter unit Amicon ultra-15, 3 kDa NMWL (Product number UFC900324, Millipore, Tullagreen, Carrigtwohill, CORK IRL)(15 min, 4000*g*, 20 °C). The concentrates were buffered (Tris, pH 9.0) and simultaneously labeled with 2 μM FP-TAMRA and 500 μM PF-biotin (6-*N*-biotinylaminohexyl isopropyl phosphorofluoridate product number B394900, Toronto Research Chemicals, North York ON Canada dissolved in DMSO) for 90 min. The sample was adjusted to 10 mM DTT heated to 55 °C, 30 min,) and alkylated (50 mM IAA, 30 min, room temperature, in the dark). Reduced samples were desalted with Zeba spin columns (Product number #89893, Thermo scientific, Rockford, IL USA). The desalting procedure was repeated with a second column.

The desalted samples were incubated for 1 h with rotation at room temperature with 20 μL protein G Sepharose beads 4 fast flow (Product number 17–0618–01, GE Healthcare SE-75184 Uppsala, Sweden) containing 40 μg of cross-linked anti-TAMRA antibody (Product number MA1–041, Thermo scientific, Rockford IL USA). The beads were collected by centrifugation (11,000*g*, 45 s). The supernatant was retained and the beads washed with PBS 0.02% Tween20.

FP-TAMRA probe-labeled enzymes were eluted from beads with 200 μL 1%TFA and centrifugation (11,000*g*, 45 s). After two cycles of affinity purification, the eluted FP-TAMRA probe-labeled enzymes from each extraction were pooled, dried in a speed-vac, dissolved (100 mM ammonium bicarbonate, 100 μL) and incubated overnight with trypsin (500 ng, 37 °C). The reaction was stopped with 4% TFA (50 μL), then frozen and dried in speed-vac; and was ready for mass spectrometry.

Unbound material from the FP-TAMRA purifications was pooled with material retained from the washing steps and concentrated to 250 μL with a centrifugal filter unit Amicon ultra-15, 3 kDa NMWL (Product number UFC900324, Millipore, Tullagreen, Carrigtwohill, CORK IRL) (55 min, 4000*g*, 20 °C). The concentrated sample was adjusted to 5 M urea mixed with 100 μL of PBS-washed streptavidin agarose resin (Product number #20353, Thermo scientific, Rockford IL USA). The sample was incubated at room temperature overnight with rotation. Beads were washed twice with 1 mL 1% SDS, 1 mL 6 M urea, 1 mL PBS and 500 μL 100 mM ammonium bicarbonate. Bound proteins were digested on bead overnight at 37 °C with 500 ng trypsin (Sequencing grade Modified Trypsin, V5111). The reaction was stopped with 50 μL 4%TFA and the beads were vortexed for 10 min and centrifuged (11,000*g*, 3 min). Supernatant was retained and the beads extracted a second time with 200 μL 0.1% TFA in acetonitrile. Pooled TFA eluents were dried on speed-vac, suspended in 500 μL of 0.5% TFA and desalted with a C18-SD extraction disc cartridge (3 M, USA, 4215SD) for analysis on 2D LC–MS/MS.

Initial experiments using samples from the same patient groups as above were used to generate two 6 mL pools per patient group. One pool from each group was labelled with either 2 μM FP-TAMRA or 500 μM PF-biotin. The individual pools were analyzed separately. The list of serine hydrolases obtained for each patient group were merged to generate Table 2.

### Nano-RPLC–MS/MS

Samples were analyzed by nano-RPLC–MS/MS using a splitless Ultra 2D Plus (Eksigent, Dublin, CA) system coupled to a high-speed Triple TOF 5600 mass spectrometer (AB SCIEX, Concord, Canada). Peptides were injected via a PepMap100 trap column (0.3 × 5 mm, 5 μm, 100 Å, Dionex, Sunnyvale, CA), and a 100 μm × 200 mm analytical column packed with 3 μm Luna C18 [2] was used prior to MS/MS analysis. Both eluents A (water) and B (98% acetonitrile) contained 0.1% formic acid as an ion-pairing modifier. The tryptic digest was analyzed in duplicate with 90 min gradient. Eluent B had a gradient from 0% to 35% over 77 min, 35% to 85% in 1 min and was kept at 85% for 5 min at a flow rate of 500 nL/min. Key parameter settings for the TripleTOF 5600 + mass spectrometer were as follows: ionspray voltage floating (ISVF) 3000 V, curtain gas (CUR) 25, interface heater temperature (IHT) 150, ion source gas 1 (GS1) 25, declustering potential (DP) 80 V. All data was acquired using information-dependent acquisition (IDA) mode with Analyst TF 1.6 software (ABSCIEX, USA). For IDA parameters, 0.25 s MS survey scan in the mass range of 400–1250 were followed by 20 MS/MS scans of 100 ms in the mass range of 100–1600 (total cycle time: 2.3 s). Switching criteria were set to ions greater than mass to charge ratio (*m*/*z*) 400 and smaller than *m*/*z* 1250 with a charge state of 2–5 and an abundance threshold of more than 150 counts. Former target ions were excluded for 5 s. A sweeping collision energy setting of 37 ± 15 eV was applied to all precursor ions for collision-induced dissociation.

The MS/MS spectra analysis was performed using X-tandem and the GPM. All proteins had ≥ 2 peptides identified and a log_10_ expectation of less than − 4. Serine hydrolases are reported based on their activities as either a metabolic or serine protease. Assignments as serine hydrolase activity are based on Uniprot [24] the MEROPs database [25] and the serine hydrolase list by Bachovchin and Cravatt [26].

### Urine PR3/PRTN3 enzyme assay

The assay conditions developed by Korkmaz et al. [27] were optimised to detect PR3/PRTN3 in urine samples. After thawing, urines were desalted using Zeba spin plates (Thermo Scientific, product number 1860080, 3747 N. Meridian road, Rockford, IL 61101 U.S.A). A 90 µL aliquot of Zeba cleaned urine was combined with reaction buffer to reach a final concentration of 50 mM HEPES buffer pH7.4, 750 mM NaCl, 0.05% IGEPAL CA-630. The reaction mixture was mixed for 2 min before aliquoting to the plate and incubating for an additional 1 h at 37 °C. The substrate, ABZ-VAD(nor)V RDRQ-EDDnp (Peptides International, SNP-3232-v) stock dissolved in 30% (v/v) *N*,*N*-dimethylformamide was diluted to 0.2 mM with 50 mM Hepes buffer, pH 7.4 and then added at a final concentration of 5 µM and the reaction was monitored at Ex/Em 320/420 nm at 37 °C for 90 min. The slopes of duplicate reactions for each sample were estimated in the 15–30 min linear reaction range after correction for any background fluorescence occurring in the same urine assayed without substrate.

### Inhibition studies

An aliquot of 90 µL urine was incubated with the 50 nM PR3/PRTN3 specific activity-based probe, Bt-Pro-Tyr-Asp-(nor)Val^P^(O-C_6_H_4_-4-Cl)_2_ [28], under assay conditions for 1 h at 37 °C before adding substrate and the assay was performed as described above. For visualization of labelled PR3/PRTN3, the inhibitor treated sample was heated in the presence of SDS sample buffer, reduced with 50 mM DTT and separated on Bolt 4 − 12% Bis–Tris plus SDS-PAGE gels. Separated proteins were transferred to a nitrocellulose membrane at 0.35 A for 1 h. After washing and blocking, membrane was incubated with 1/5000 Alexa Fluor^®^ 488-streptavidin before acquiring images.

### Western blot

Either 30 µL of urine or 20 ng of purified PR3/PRTN3 (Athens Research and Technology catalogue number 16-14-161820) were separated by SDS-PAGE and then transferred to nitrocellulose membrane. The membrane was washed wit 0.1% Tween 20 in PBS, blocked with washing solution containing 5% skim milk then with 0.1 µg/mL antibody to human PR3/PRTN3 (R&D, catalogue number MAB6134). The membrane was washed 3 times for 10 min in 0.1% Tween 20 in PBS and incubated with a peroxidase conjugated anti-mouse IgG (Sigma SAB3701066) at 50 ng/mL. The blot was washed 3 times for 10 min in 0.1% Tween 20 in PBS developed with ECL western blotting detection reagent (Amersham catalogue number RPN2106). The blot was photographed using an Amersham 680 imager.

## Results

### Activity-based protein profiling of subclinical and clinical TCMR

The patient characteristics for ABPP analysis are described in Table 1. None of the patients had end-stage renal disease from ANCA vasculitis and none of the biopsies showed evidence of post-transplant ANCA disease. All patients had a minimal degree of proteinuria (< 0.5 g/day).

**Table 1:** Subclinical rejection cohort patient characteristics, used for activity-based protein profiling Adapted from Ho et al. [18]

Patient characteristic	All n = 18	Normal transplant n = 6	Subclinical TCMR Banff ≥ 1A n = 6	Clinical TCMR Banff ≥ 1A n = 6
At transplant				
Recipient age (years)	50.6 ± 9.8	48.7 ± 5.1	52.2 ± 4.4	50.9 ± 16.5
Recipient sex (male)	15 (83.3)	6 (33.3)	4 (22.2)	5 (27.8)
Recipient race (Caucasian)	17 (94.4)	5 (27.8)	6 (33.3)	6 (33.3)
Cause of end-stage renal disease				
Diabetes	4 (22.2)	3 (50.0)	0	1 (16.7)
Glomerulonephritis	7 (38.9)	2 (33.3)	3 (50.0)	2 (33.3)
Polycystic kidney disease or congenital	5 (27.8)	1 (16.7)	2 (33.3)	2 (33.3)
Hypertension	1 (0.06)	0	0	1 (16.7)
Drug toxicity (lithium)	1 (0.06)	0	1 (16.7)	0
HLA mismatch	3.78 ± 1.4	3.17 (1.8)	4.17 (1.5)	4.0 (0.9)
Panel reactive antibody (PRA)	0.78 ± 2.3	2.33 ± 3.7	0	0
Donor type (living)	12 (66.7)	3 (16.7)	3 (16.7)	6 (33.3)
Donor age (years)	39.6 ± 13.2	38.8 ± 14.8	38.3 ± 17.7	41.5 ± 7.2
Induction therapy (none/basiliximab/thymoglobulin)	13/4/1	5/0/1	4/2/0	4/2/0
Post-transplant				
Maintenance therapy: tacrolimus, mycophenolate mofetil and prednisone	14 (77.8)	6 (33.3)	4 (22.2)	4 (22.2)
Delayed graft function (dialysis in the 1st week post-transplant)	0	0	0	0
Biopsy time (weeks post-transplant)	15.7 ± 10.0	20.8 ± 12.0	14.3 ± 9.0	11.8 ± 7.8
Banff i score	1.6 ± 1.0	0.3 ± 0.8	2.2 ± 0.4	2.2 ± 0.4
Banff t score	1.7 ± 1.3	0.5 ± 1.2	2.7 ± 0.5	1.8 ± 1.0
Banff v score	0.2 ± 0.7	0	0	0.7 ± 1.2
Banff g score	0.2 ± 0.7	0	0	0.5 ± 1.2
Banff ci score	0.4 ± 0.8	0.3 ± 0.5	0.5 ± 0.5	0.5 ± 1.2
Banff ct score	0.6 ± 0.6	0.7 ± 0.5	0.5 ± 0.5	0.7 ± 0.8
Banff cv score	0.5 ± 0.9	0.4 ± 0.9	0.3 ± 0.5	0.75 ± 1.5
Banff cg score	0.1 ± 0.3	0.2 ± 0.4	0	0.2 ± 0.4
Serum creatinine at biopsy (µmol/L)	137.2 ± 45.4	125.4 ± 25.6	113.0 ± 44.1	173.2 ± 44.2
MDRD eGFR at biopsy (mL/min)	54.5 ± 18.8	59.4 ± 15.7	64.0 ± 19.1	40.1 ± 14.3
Total urine protein (g/day)	0.19 ± 0.2	0.15 ± 0.1	0.14 ± 0.07	0.28 ± 0.3

Data are reported as mean ± SD, or total count (total %)

Other maintenance immunosuppression was triple therapy with cyclosporine, mycophenolate mofetil & prednisone

The serine hydrolase probe reactive species in urine samples from individual patients with renal transplants displaying biopsy proven normal, subclinical, or rejection histological status were compared by SDS-PAGE. The gels were processed, stained and imaged in a standardised fashion in an effort to minimize any potential experimental introduced intensity biases between samples. The same gels were also stained for protein to compare protein input levels and patterns.

**Fig. 1:**
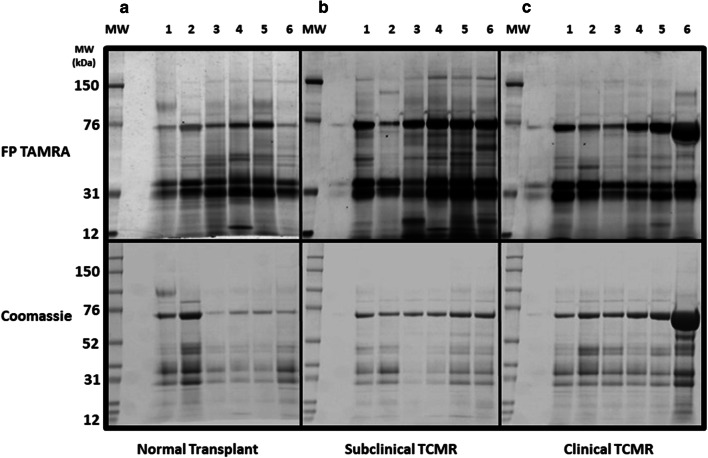
Serine hydrolase activity-based protein profiling comparison of kidney transplant rejection. Urines from biopsy confirmed. **a** Normal transplant. **b** Subclinical rejection and **c** clinical T-cell mediated rejection patients were labelled with (FP)-TAMRA and the proteins were separated by SDS PAGE. The (FP)-TAMRA labelled proteins were visualised (upper panel) and the same gels were stained with Coomassie blue to visualise total protein. Each lane contains a sample from a different patient. Molecular weight marker positions are indicated to left of each panel

There was some inter-individual and inter-group variability but the overall urine activity patterns in all groups were quite similar (Fig. 1, upper panels). The subclinical rejection urines displayed some differences in the apparent intensity and diversity of labelling relative to samples from patients with normal transplant function or those undergoing clinical rejection. The labelling of the subclinical rejection samples was particularly intense in the 27–35 kDa and 70 kDa gel regions. These differences did not appear to be a consequence of increased levels of proteins in these regions (Fig. 1, compare upper and lower panels). There also appeared to be several potentially unique lower intensity species in the 31-76 k Da regions of the subclinical gels. These results suggested that some of the activity changes detected by the (FP)-TAMRA probe were due to qualitative as opposed to quantitative changes. Overall the clinical rejection urines had less diverse patterns of labeling compared to normal transplant and subclinical rejection urines (Fig. 1, upper panels).

A limitation of the gel-based approach is that it does not identify the enzymes visualised in the gels. Furthermore a single band region may not necessarily be reflective of the activity of a single enzyme species. These issues made it critical to identify the active enzyme species in the samples.

### Identification of active urinary serine hydrolases in subclinical and clinical rejection

Initial experiments using separate probe labelled 6 mL pools from each patient group had resulted in low sample recoveries. We therefore opted to use a larger sample volume and to sequentially label the samples with the both probes as previous studies had shown that they provided some complementarity in labelling of active serine hydrolase enzymes [29]. The labelled proteins were affinity-purified and analysed by mass spectrometry (Additional file 1: Figure S1, Additional file 2: Table S3 and Additional file 3: Table S4).

Twenty-nine active urine serine hydrolases were identified in subclinical rejection patients with 7 metabolic hydrolases and 22 serine proteases. Twenty-seven active urine serine hydrolases were identified in clinical rejection patients with 6 metabolic hydrolases and 21 serine proteases. Alpha/beta hydrolase domain-containing protein 14B (ABHD14B), lactotransferrin (LTF), and proteinase 3 (PRTN3, aka PR3) were only observed in the subclinical rejection samples ([Table 2). Neurotrypsin (PRSS12) was only identified in the clinical rejection urine pool sample (Table 2).

**Table 2:**
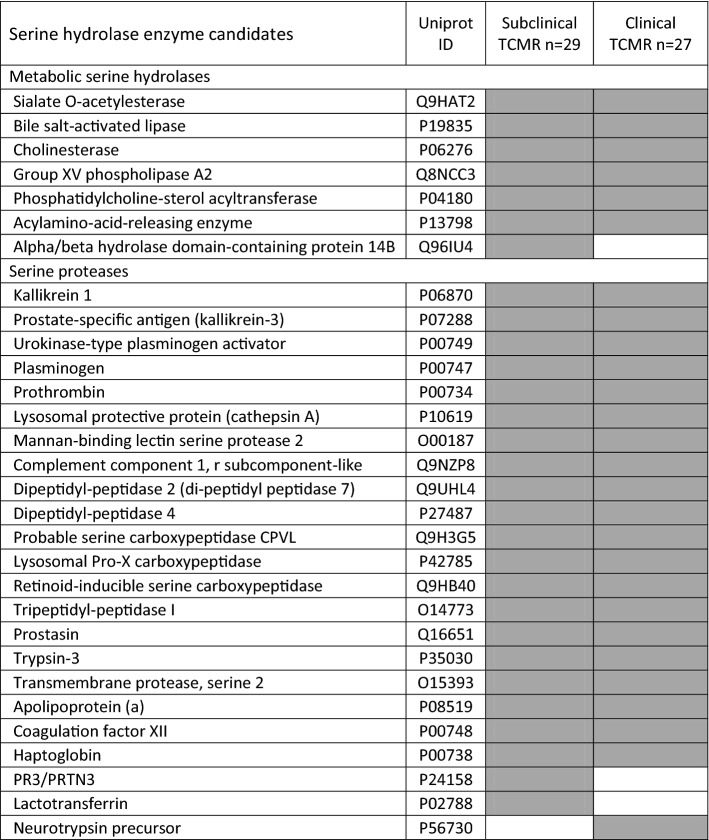
Enzyme candidates identified with activity-based protein profiling

White: not detected. Grey: Protein detected with ≥ 2 peptides AND log(e) ≤ − 4

Proteinase 3 was selected for further in-depth evaluation. This decision was in part based upon on the availability of a well characterised specific assay and the fact that this enzyme activity is not an expected normal urine. Indeed the presence of PR3/PRTN3 or related neutrophil protease activities in normal urine is the basis for a dipstick based assay indicative of potential urinary tract infection. Our previous ABPP studies had suggested that PR3/PRTN3 activity if present was at low levels in the urine of healthy non-transplant individuals [30]. Also previous ABPP studies had failed to detect PR3/PRTN3 activity in the urine of adult cardiac surgery patients independent of whether they developed post-operative acute kidney injury [31].

### Optimization of a quantitative urine PR3/PRTN3 activity assay

**Fig. 2:**
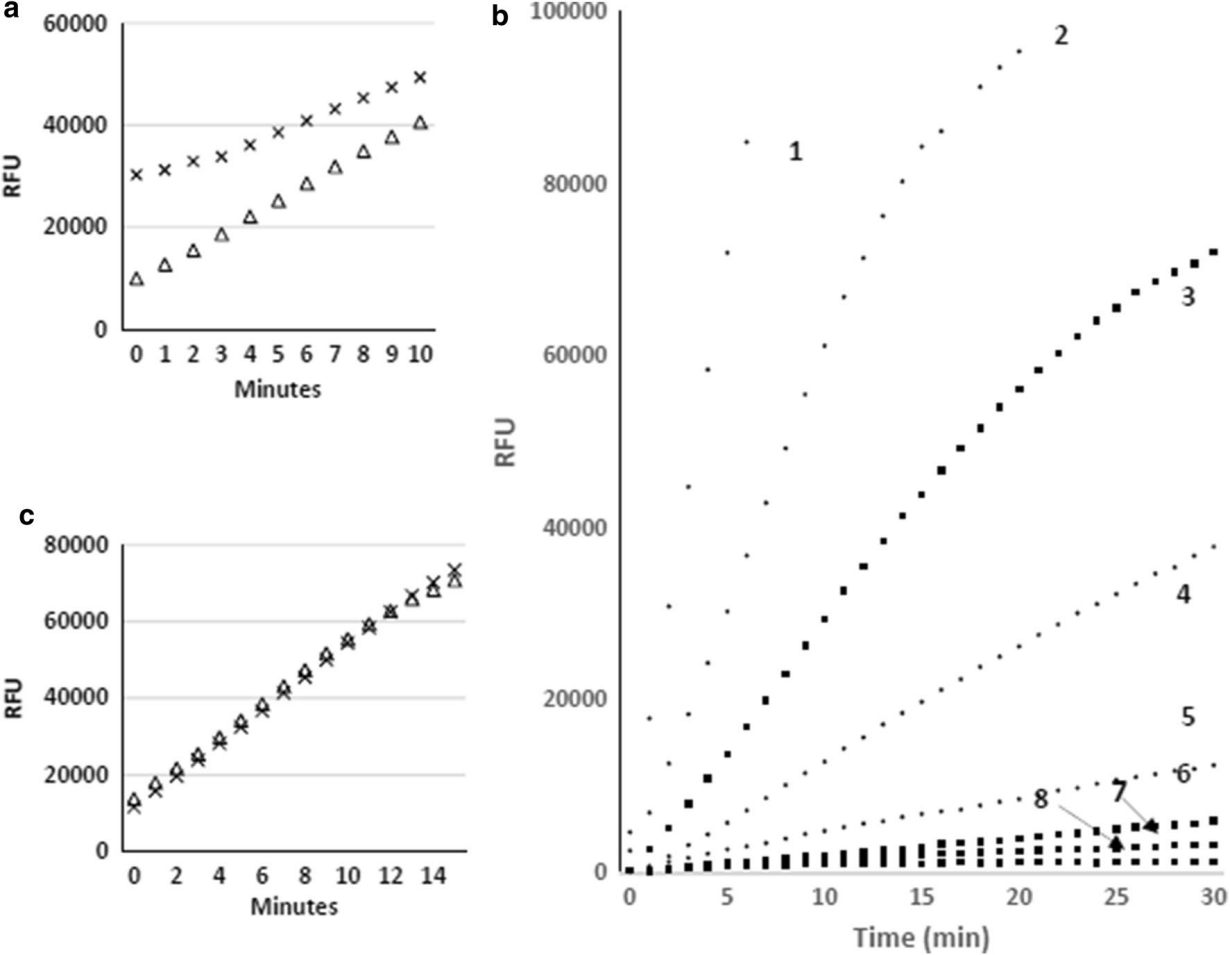
Optimization of a quantitative PR3/PRTN3 activity assay for urine. **a** Urine from a normal healthy donor was either untreated (**X**) or passed through a Zeba plate (white-up pointing triangle) and after which 4 nM of purified proteinase 3 was added and assayed. Note increased slope and linearity of activity in the Zeba passaged urine. **b** The activities of the indicated amounts of purified PR3/PRTN3 were added to an in inactive urine and assayed: (1) 8 nM, (2) 4 nM, (3) 2 nM, (4) 1 nM, (5) 0.5 nM, (6) 0.25 nM, (8) 0.125 nM, (8) 0.0625 nM. **c** The effect of repeated freezing and thawing of purified PR3/PRTN3 in urine on enzyme activity after 1 freeze/thaw (**X**) or 7 freeze/thaw cycles (white-up pointing triangle)

The activity of exogenously added PR3/PRTN3 was readily demonstrable in urine (Fig. 2a). It was initially noted that urines often contained variable but significant levels of background fluorescence which reduced the assay sensitivity, linearity and dynamic range. This fluorescence was significantly reduced by passage of the urine samples through Zeba spin plates prior to the assay (Fig. 2a). Although there often appeared to be a modest increase in the PR3/PRTN3 activity after this treatment, this was not consistently observed suggesting that endogenous activity was not masked by inhibitors in the urine.

Under our current conditions the assay was linear for more than 30 min at enzyme concentrations below 1 nM. However, at enzyme concentrations above this initial linearity was lost due to detector saturation. The assay was highly reproducible with a CV of less than 5%. These assay conditions allowed for the quantitation of 62.5–250 pM of enzyme in 40 µL urine corresponding to ~ 20 femtomoles of active enzyme (Fig. 2b). The activity of PR3/PRTN3 was stable for at least 7 freeze–thaw cycles suggesting the enzyme could be confidently monitored in biobanked urine samples (Fig. 2c).

### Characterization of PR3/PRTN3 activity in urine

**Fig. 3:**
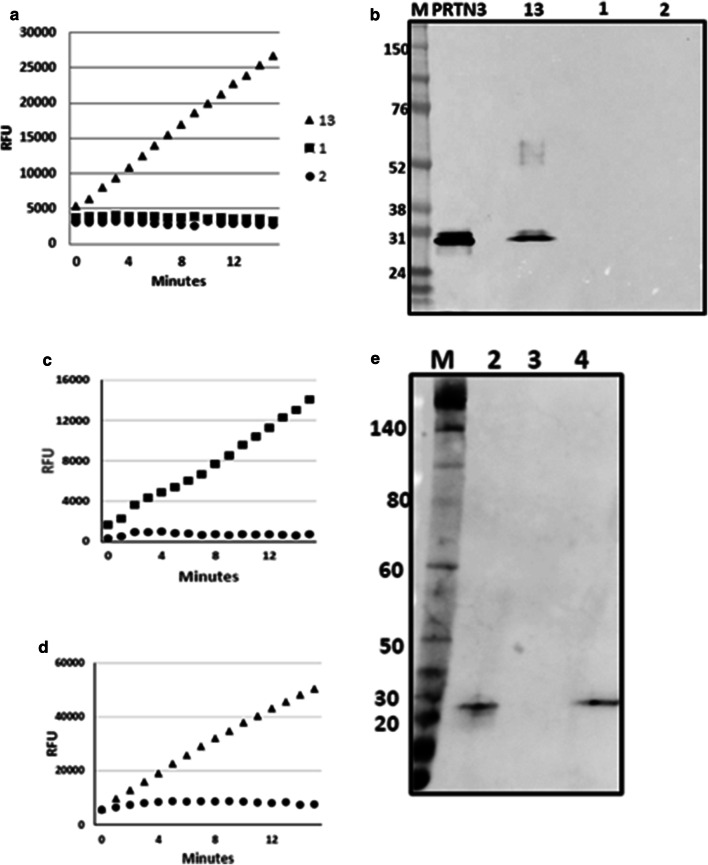
Characterization of PR3/PRTN3 activity in subclinical rejection urine. **a** The PR3/PRTN3 activity in urines from three patients displaying subclinical rejection at the time of sample collection. **b** Samples of the same urines as **a**) (lanes 1, 2, 13) or purified enzyme (lane PRTN3) were SDS PAGE separated, blotted and probed with anti- PR3/PRTN3. Molecular weight markers (lane M). **c** Purified proteinase 3 or **d** a proteinase 3 active subclinical urine were treated with DMSO alone (black square, black-up pointing triangle) or the PR3/PRTN3 specific inhibitor Bt-Pro-Tyr-Asp-(nor)Val^P^(O-C_6_H_4_-4-Cl) 2 dissolved in DMSO (black circle). **e** Aliquots of the inhibitor treated samples in **c** and **d** or an untreated sample of the same urine were separated by SDS-PAGE, blotted and probed with Alexa fluor™ 488-streptavidin. Lanes M) Molecular weight markers; (1) Inhibitor-labelled purified PR3/PRTN3; (2) Untreated urine; lane 4) Inhibitor-treated subclinical rejection urine (same urine as in **d**)

Endogenous PR3/PRTN3 activity was detected in the urine samples of some patients undergoing subclinical rejection at the time of sample collection (Fig. 3a). Western blot analysis of these urines demonstrated the presence of the enzyme in the active but not in the inactive urine samples demonstrating a correlation between the presence of PR3/PRTN3 and assay activity (Fig. 3b).

The activity of purified PR3/PRTN3 (Fig. 3c) and the endogenous activity in subclinical urine (Fig. 3d) were completely blocked by the selective inhibitor, Bt-Pro-Tyr-Asp-(nor)Val^P^(O-C_6_H_4_-4-Cl)_2_, [28]. This inhibitor is biotinylated allowing for the visualisation on blots of the proteins that it reacted with. A single band with the same mobility as inhibitor treated PR3/PRTN3 was detected on the SDS PAGE separated samples (Fig. 3e). These results suggested that PR3/PRTN3 was responsible for all of the assay activity detected in the urine samples.

### Urine PR3/PRTN3 activity in kidney transplant patients with subclinical and clinical rejection

**Fig. 4:**
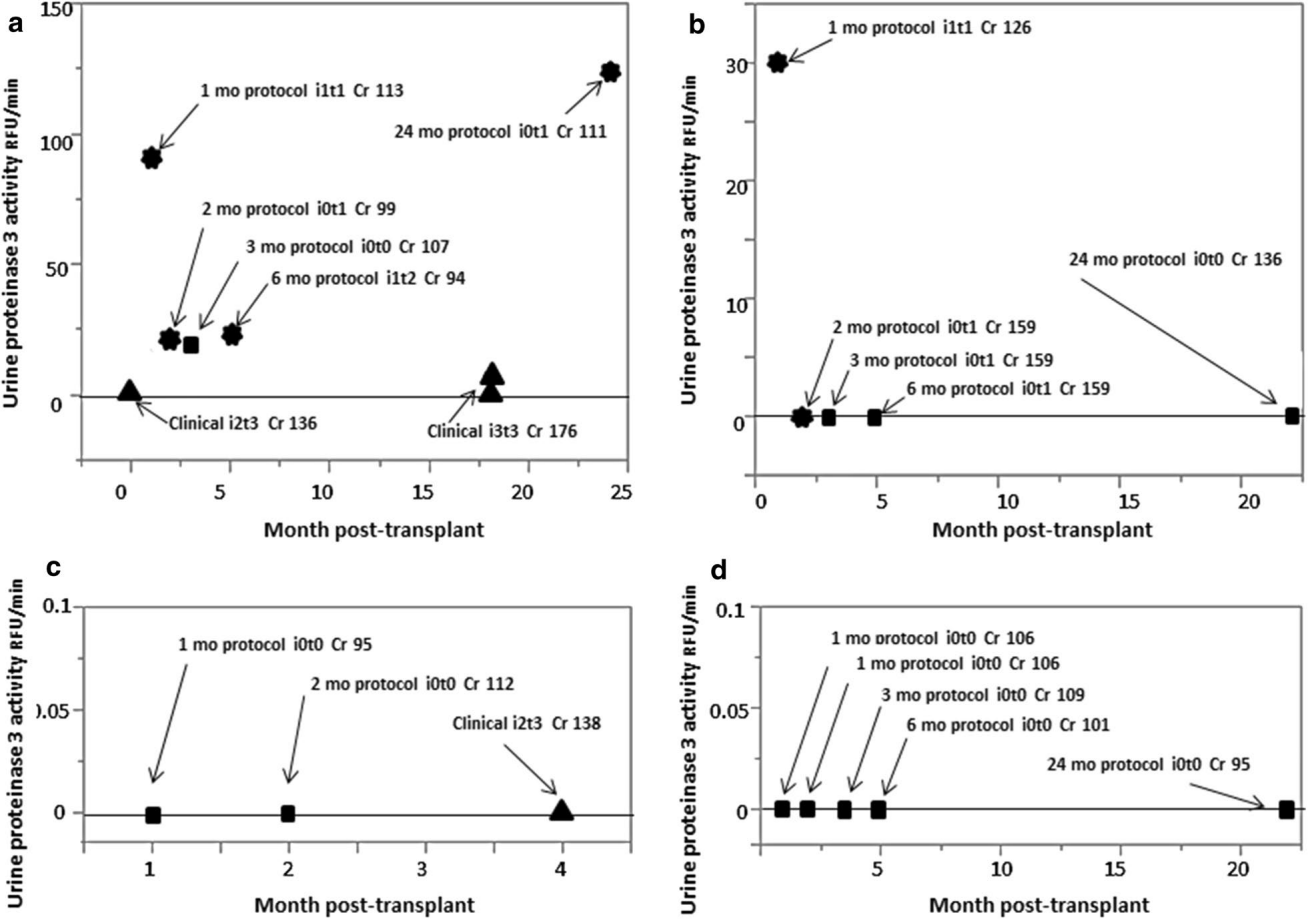
Urine proteinase-3 activity over time in kidney transplant patients with serial histology. Four kidney transplant patients had serial protocol (surveillance) biopsies at pre-specified times post-transplant or as clinically indicated. Urine samples taken at these time points were evaluated for PR3/PRTN3 activity. **a–d** Serial protocol biopsies demonstrated a trend towards low urine proteinase-3 activity with stable transplants (black square, normal histology and stable graft function, Banff i0t0) and clinical T-cell mediated rejection Banff ≥ 1A (black-up pointing triangle, clinical Banff ≥ 1A rejection, Banff i2–3 t2–3). Elevated urine PR3/PRTN3 activity was detected in a significant proportion of samples from kidneys with low grade subclinical inflammation ( , borderline subclinical inflammation, Banff i0–1 t1–2)

The classification of transplant rejection status for a given individual may not be fixed over time (e.g. rejection vs stable). The histological analysis of biopsies of a transplanted kidney offers a basis for defining these different rejection states in an individual. A sequential series of urine samples from four patients from a larger prospective cohort [32, 33] who had undergone serial protocol (surveillance) biopsies or clinically indicated biopsies were examined to determine if there was a relationship between graft status at the time of urine sample collection and urinary proteinase 3 activity (Fig. 4). All biopsies were scored using the Banff schema by a single pathologist.

The majority (10 of 11) of samples from patients with stable transplants at the time of urine collection (i.e. normal histology and stable graft function, Banff i0t0) had little or no PR3/PRTN3 activity (Fig. 4b–d). Similarly samples from patients who received clinically indicated biopsies which indicated TCMR (clinical Banff ≥ 1A rejection, Banff i2–3 t2–3) at the time of sample collection displayed either low or no detectable levels of PR3/PRTN3 activity (Figs. 4a, c). However 5 of 6 samples from patients who displayed low grade subclinical inflammation (borderline subclinical inflammation, Banff i0–1 t1–2) at the time of urine collection had elevated levels of PR3/PRTN3 activity in their urine samples (Fig. 4a, b).

**Fig. 5:**
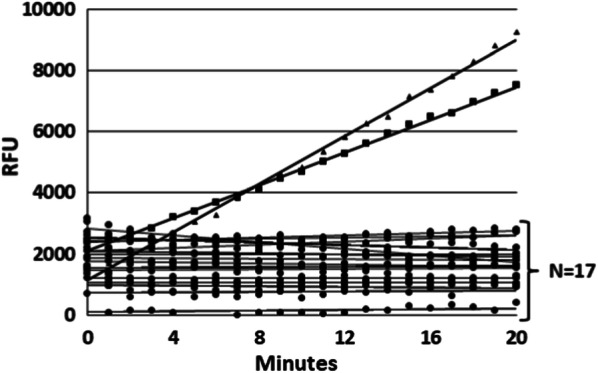
PR3/PRTN3 activity in normal urine samples. Samples from 19 healthy normal donors were assayed for PR3/PRTN3 activity

Analysis of PR3/PRTN3 activity in urine from a different series of transplant patients from those tested above detected activity in some but not all patients (Additional file 3: Figure S2). Activity was not detected in any of the samples from patients with normal renal histology. However activity was detected in a third of samples tested from patients with subclinical or clinical rejection at the time of sample collection. The PR3/PRTN3 activity in the urines from patients with subclinical rejection was higher than that observed in patients undergoing clinical rejection. PR3/PRTN3 activity was also detected in 2 of 19 urines from normal healthy controls (Fig. 5) suggesting that the frequency of active samples in these donors was lower than that observed in the transplant patients.

## Discussion

This study provides evidence that there a quantitative or qualitative differences between the active serine hydrolases in the urines of patients undergoing subclinical or clinical rejection. The majority of the active enzymes detected in urine were observed in both patient groups. However the activities of ABHD14B, LTF, PR3/PRTN3 and PRSS12 were differentially observed between the two patient groups. The presence of active PR3/PRTN3 in urine was confirmed by western blot and activity was demonstrated using highly specific substrate and inhibitor. Although PR3/PRTN3 activity was detected in a significant proportion of urine samples from patients with low-grade subclinical inflammation activity was also detected at a lower frequency in urine samples from patients undergoing clinical rejection or from some normal healthy donors. These results indicated that PR3/PRTN3 urinary activity was not a unique feature of subclinical rejection. The results also demonstrated that PR3/PRTN3 activity can vary over time within a single transplant recipient (Additional file 2).

Thirty active serine hydrolases were identified in urines collected from patients displaying subclinical or clinical rejection. However, the majority of these enzyme activities were detectable in both groups indicating that these enzymes not unique either transplant state. These observations were consistent with the in-gel analysis of probe labelled proteins from the two groups which indicated minor qualitative differences but significant qualitative differences in activity. The differentially active enzymes identified in the analysis have not previously been characterised in the context of transplantation. ABHD12B was originally isolated in a yeast two hybrid screen for proteins interacting with the histone acetyltransferase activity associated domain of CCG1 [34]. Recently ABHD14B has been shown to possess acetyl transferase activity [35]. Initial western blot based distribution studies in the mouse indicated that the enzyme is highly expressed in the liver and kidney. Lactotransferrin is a multi-functional protein with diverse antimicrobial and immunoregulatory functions including a weak serine hydrolase activity involved in antimicrobial resistance [36, 37]. PSS12 which was only detected in the clinical rejection urine sample cleaves agrin to generate a C terminal 22 kDa fragment [38]. The appearance of this fragment in urine is a predictor of loss of kidney function. Significantly PSS12 is the only enzyme known to be capable of generating this fragment suggesting that the appearance of activity may be relevant in the context of loss of renal function during transplant rejection. It is also noteworthy that many of the other enzyme candidates identified in the present study have not previously been characterized as rejection-associated transcripts [39]. These results highlight the complementary value of evaluating changes in enzymatic activity in conjunction with quantitative compositional analysis of proteins [22] (Fig. 4).

PR3/PRTN3, also known as myeloblastin, azurophil granule protein-7 or p29b, is a serine hydrolase with broad specificity against a number of extracellular matrix proteins including elastin, fibronectin, laminin, vitronectin, and several collagen types [40–42]. PR3/PRTN3 can also proteolytically activate TNF-α and extracellular IL-1β [43, 44]. It has been suggested that PR3/PRTN3 activity may play a protective role in the glomerular basement membrane by cleavage of von Willebrand factor helping to prevent thrombus formation [45]. There is also some evidence to suggest that PR3/PRTN3 activity may contribute to the pathology of other disease states [41, 46]. Proteinase 3 was selected for further analysis because of the reported low levels or absence of activity this enzyme in normal urines and the availability of a specific assay.

The assay was adapted for the analysis of urine which permitted the large scale quantitative assessment of PR3/PRTN3 using small sample volumes. This is a particularly relevant factor in the case of limiting quantities of clinically relevant samples. Although there appeared to be some relationship between the presence of subclinical rejection at the time of sample collection and increased frequencies and higher activities of PR3/PRTN3 compared with the other patient groups this was not absolute. The histological analysis of serial biopsies and the assay of urines collected at the same time for PR3/PRTN3 indicated a strong but not absolute correlation between subclinical inflammation and the presence of activity. It was also apparent that the enzyme activity could vary over time much as the inflammatory status of a transplant could. A small proportion of samples from healthy normal controls had some of the highest activities detected.

Neutrophils are a major source of PR3/PRTN3, elastase, and cathepsin G which are stored in azurophil granules [40]. Urinary tract infection can result in the recruitment of neutrophils and the release these granules. This appears unlikely to be the case in the transplant patients as all patients were negative for evidence of urinary tract infection at the time of biopsy and urine collection. Proteinuria can be another possible confounder as PR3/PRTN3 levels are increased in urinary extracellular vesicles isolated from diabetic patients with normo- or microalbuminuria [48]. However, patients in the present study had minimal proteinuria (< 0.5 g/day), suggesting that this was not significant a contributing factor. It was not possible to rule out the possibility of subclinical urinary tract infection in the normal donors as cultures were not been performed on these samples.

Neutrophil-rich infiltrates are uncommon during rejection and up to half of infiltrating leukocytes in renal allograft rejection are macrophages [47]. Thus it is unclear what the cellular sources or processes are that contribute to the appearance of PR3/PRTN3 in urine in the transplant patients. However the current observations raise the possibility that another cell type, possibly infiltrating macrophages, may be the source of PR3/PRTN3 activity [40]. However this remains to be determined.

One intriguing possibility is that the elevated serine hydrolase activity state of subclinical rejection detected on ABPP gels may be a dynamic and reversible state reflecting processes that are occurring in the graft. Interestingly, the clinical rejection ABPP gels suggested less diverse enzyme activities compared to normal transplants. This is notable since normal urine serine hydrolase activities in healthy individuals include luminal regulators of electrolyte homeostasis, such as tissue kallikrein and plasmin [30]. Loss of such homeostatic mechanisms may contribute to loss of graft function during clinical rejection. These data are consistent with a human acute kidney injury model, wherein serine hydrolase activity changes precede or accompany loss of renal function [31]. Taken together, further exploration of these serine hydrolase candidates and their differential enzyme activities may provide fresh insight into the analysis histologically identical but functionally divergent graft phenotypes associated with subclinical and clinical rejection.

The present studies specifically focused on the differences between clinical and subclinical transplant rejection patients. Thus the data does not necessarily indicate that any of the enzyme activities detected are unique to the rejection process. Pooled urine samples were used for the ABPP isolation because of the limitations on the amount of clinical material available. This introduced the potential for a few samples to skew the results and limits the ability to unequivocally assign the presence of a given enzyme as a general feature of a patient group. However the approach does provide a rational basis for the selection of candidate enzymes for the application of quantitative assays to test such correlations. Although there appeared to be some differences in ABPP gel patterns and intensities there are clear limitations on the interpretation of such results as it is difficult to quantitatively compare between gels and also a single active region on a gel may be the result of several active species. These considerations make it imperative to identify and quantitatively assay individual enzyme species.

## Conclusions

This study suggests a potential relationship between the urinary PR3/PRTN3 activity and low grade subclinical rejection. The availability of a highly sensitive assay for PR3/PRTN3 offers a basis for the high throughput comparative analysis of large scale clinical cohorts required to further delineate these critical questions. The cellular origin of PR3/PRTN3 in these patients also remains to be determined. Although this study evaluated urinary PR3/PRTN3 activity, these results do not preclude the possibility that the other serine hydrolase enzyme candidates identified by ABPP may also play critical roles in pathophysiology of subclinical and clinical rejection.

## Supplementary information

**Additional file 1: Figure S1.** Affinity purification of FP-TAMRA/PF-biotin labelled urines proteins from patients undergoing clinical (upper panels) or subclinical rejection (lower panels) rejection. Samples were treated with both probes and sequentially affinity purified using an anti-FP TAMRA antibody column followed by a streptavidin affinity column. **A)** Fractions stained for protein. **B)** The patterns of FP TAMRA labeling of the same gels as is A).The lanes represent: (1) Concentrated urines activity-probe labeled, reduced and alkylated; (2) Sample after Zeba treatment; (3) Flow-through i.e. proteins that did not bind to the affinity column; (4) 1st elution from the beads; (5) 2nd elution; (6) Material retained by beads and not eluted.). **C)** The flow through protein from the FP TAMRA affinity column (i.e. material analysed in lane 3) was passed through a streptavidin column. Lanes (7) Flow-through (8;) 1st elution from the beads; (9) 2nd elution; (10) material retained by beads and not eluted. The images demonstrate the enrichment of probe labelled materials in fractions with significantly lower amounts of protein.

**Additional file 2.** List of all proteins identified by mass spectromety in the urine samples from patients undergoing clinical rejection.

**Additional file 3.** List of all proteins identified by mass spectromety in the urine samples from patients undergoing subclinical rejection.

**Additional file 4: Figure S2.** Samples from renal transplant patients (6 patients per group) with the indicated graft status at the time of urine collection were assayed for PR3/PRTN3 activity.

## Data Availability

The datasets used and/or analysed during the current study are available from the corresponding author on reasonable request.

## Abbreviations

ABPP: Activity-based protein profiling
ANCA: Anti-neutrophilic cytoplasmic antibodies
FP: Fluorophosphonate
PF: Phosphorofluoridate
PR3/PRTN3: Proteinase 3
REB: Research ethics board
SCR: Subclinical rejection
SDS-PAGE: Sodium dodecyl sulfate polyacrylamide gel electrophoresis
TCMR: T cell mediated rejection
