# Supplementary figures and images for: Activity-based protein profiling guided identification of urine proteinase 3 activity in subclinical rejection after renal transplantation
Source: Clin Proteomics. 2020 Jun 16;17:23. doi: 10.1186/s12014-020-09284-9 (PMC7296916; doi:10.1186/s12014-020-09284-9)

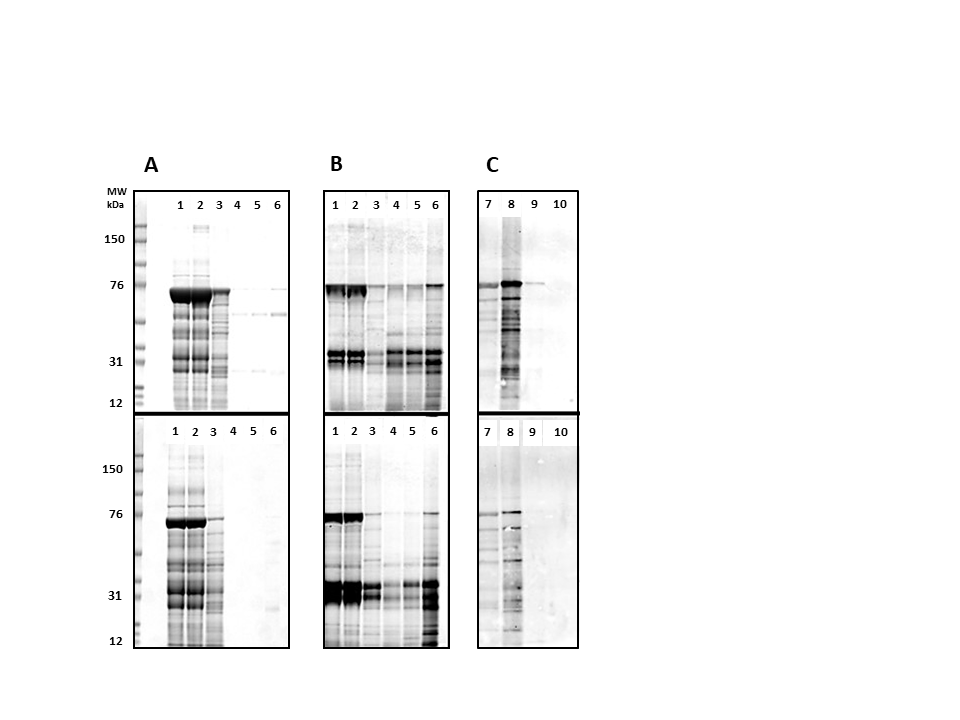

Supplement: Supplementary file 1 — Additional file 1: Figure S1. Affinity purification of FP-TAMRA/PF-biotin labelled urines proteins from patients undergoing clinical (upper panels) or subclinical rejection (lower panels) rejection. Samples were treated with both probes and sequentially affinity purified using an anti-FP TAMRA antibody column followed by a streptavidin affinity column. A) Fractions stained for protein. B) The patterns of FP TAMRA labeling of the same gels as is A).The lanes represent: (1) Concentrated urines activity-probe labeled, reduced and alkylated; (2) Sample after Zeba treatment; (3) Flow-through i.e. proteins that did not bind to the affinity column; (4) 1st elution from the beads; (5) 2nd elution; (6) Material retained by beads and not eluted.). C) The flow through protein from the FP TAMRA affinity column (i.e. material analysed in lane 3) was passed through a streptavidin column. Lanes (7) Flow-through (8;) 1st elution from the beads; (9) 2nd elution; (10) material retained by beads and not eluted. The images demonstrate the enrichment of probe labelled materials in fractions with significantly lower amounts of protein. [file 12014_2020_9284_MOESM1_ESM.tif]

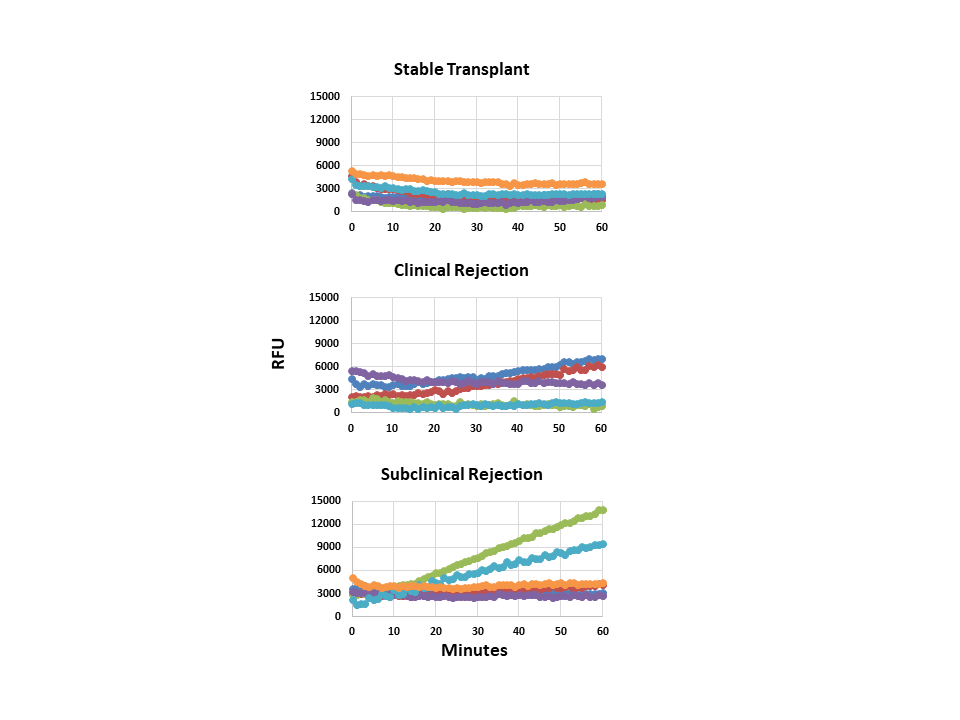

Supplement: Supplementary file 4 — Additional file 4: Figure S2. Samples from renal transplant patients (6 patients per group) with the indicated graft status at the time of urine collection were assayed for PR3/PRTN3 activity. [file 12014_2020_9284_MOESM4_ESM.tif]
